# Emotional Distress and Academic Stressors Among Medical Students at a Medical College in Oman

**DOI:** 10.1192/bjo.2026.11199

**Published:** 2026-06-30

**Authors:** Salim Al-Huseini, Mohammed Qutishat, Firdous Jahan, Mandhar Almaqbali

**Affiliations:** 1 Al Masarra Hospital, Ministry of Health, Muscat, Oman; 2 College of Nursing, Sultan Qaboos University, Muscat, Oman; 3 College of Medicine National University, Sohar, Oman; 4 Ministry of Health,Sohar, Oman

## Abstract

**Aims:**

Emotional disorders are increasingly prevalent among medical students, significantly affecting their well-being and academic performance due to high levels of academic pressure. This study aims to assess emotional distress at Oman Medical College, addressing a gap in understanding mental health within this demographic and underscoring the need for targeted interventions.

**Methods:**

Conducted from April to May 2025, this descriptive cross-sectional study utilized standardized surveys to evaluate emotional distress among medical students at the National University of Science and Technology in Oman. Data were collected through an online questionnaire from a convenience sample of 400 students, employing the General Health Questionnaire (GHQ-12) for mental health assessment. Statistical analysis was performed using SPSS to determine significance.

**Results:**

Of the 400 students invited, 336 completed the survey, yielding a response rate of 84%. The average age of participants was 21.6 years, with the majority identifying as female (89.6%) and Omani nationals (78.2%). Emotional distress was reported by 73% of respondents, with exam-related stress identified as a key contributing factor. Notably,students who received strong parental support exhibited significantly lower levels of emotional distress (p <0.05).

**Conclusion:**

This study reveals a high prevalence of emotional distress among medical students in Oman, closely linked to academic stressors and mitigated by familial support. These findings highlight the urgent need for tailored mental health programmes and resilience-building interventions within medical education in Oman.

